# Iridoschisis—A Systematic Review

**DOI:** 10.3390/jcm9103324

**Published:** 2020-10-16

**Authors:** Barbara Pieklarz, Emil Tomasz Grochowski, Emil Saeed, Patryk Sidorczuk, Zofia Mariak, Diana Anna Dmuchowska

**Affiliations:** Department of Ophthalmology, University Teaching Hospital of Bialystok,15-276 Bialystok, Poland; emil.grochowski@umb.edu.pl (E.T.G.); emilsaeed1986@gmail.com (E.S.); patryk.sidorczuk@gmail.com (P.S.); mariakzo@umb.edu.pl (Z.M.); diana.dmuchowska@umb.edu.pl (D.A.D.)

**Keywords:** iridoschisis, iris degeneration, free-floating iris tissue, angle-closure glaucoma, rare ocular disease

## Abstract

Iridoschisis is a rare condition defined as a separation of the anterior iris stroma from the posterior stroma and muscle layers. In this paper, we review current data about the epidemiology, pathophysiology, clinical characteristics and differential diagnoses of this condition and discuss the specificity of surgical treatment of concomitant ocular diseases in iridoschisis patients. Iridoschisis may pose a challenge for both an ophthalmologist in an outpatient setting and an ophthalmic surgeon. Glaucoma, primarily angle-closure glaucoma, is the most often described condition concomitant to iridoschisis. Other ocular abnormalities found relatively often in iridoschisis patients include cataract, lens subluxation and corneal abnormalities. Special attention has been paid to potential complications of cataract surgery and prevention thereof. Beside addressing the practical aspects, we point to discrepancies and suggest topics for further investigation.

## 1. Introduction

Iridoschisis is a rare condition which may pose a challenge for both an ophthalmologist in an outpatient setting and an ophthalmic surgeon. The aim of this paper was to review current data about the epidemiology, pathophysiology, clinical characteristics and differential diagnoses of iridoschisis, and to discuss the specificity of surgical treatment of concomitant ocular diseases in patients with this condition. Moreover, conditions that may coexist with iridoschisis and require ophthalmologists’ consideration have been reviewed extensively. Special attention has been paid to potential complications of cataract surgery and prevention thereof in iridoschisis patients. Finally, the data about the occurrence, characteristics and treatment of glaucoma in patients with iridoschisis have been summarized, and possible directions for future research have been outlined.

## 2. Method of Literature Search

**Figure 1:**
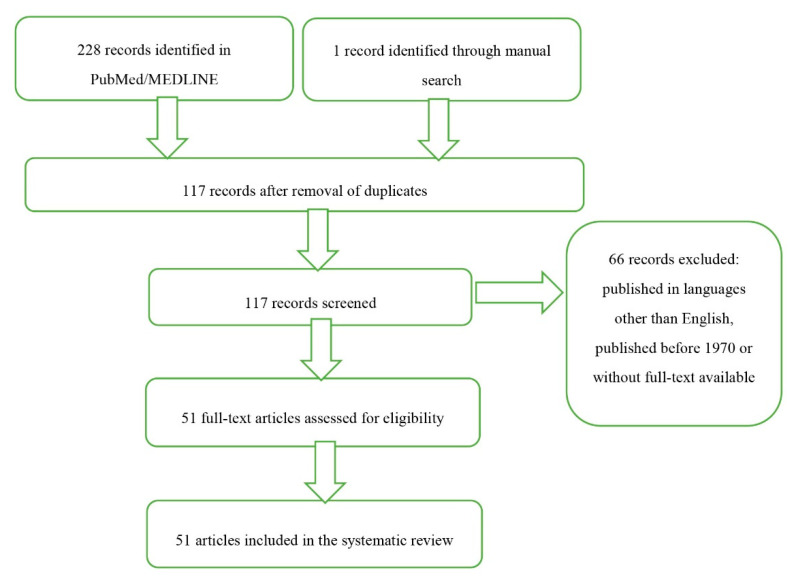
PRISMA flow chart documenting the selection of articles for the review.

An extensive search of PubMed and MEDLINE databases has been carried out for the articles published before 1 September 2020 and containing the term “iridoschisis”. A total of 111 and 117 potentially eligible articles were identified in the two databases, respectively. Additionally, one record was identified through a manual search. The list of identified records contained 28 papers published during the last ten year, among them 22 that were eventually included in this review. While no time criterion has been applied, some papers published before 1970 were not considered, whereas other older articles were included because of their historical value. We decided to focus on the most recent data. The search was limited mostly to articles published in English, but also two French papers were included in the review for their informative value (Figure 1).

## 3. Definition

Iridoschisis is a rare condition defined as a separation of the anterior iris stroma from the posterior stroma and muscle layers. In iridoschisis patients, the iris strands float in the aqueous humor and create a “shredded wheat” appearance [1]. The term iridoschisis (iris splitting) was first introduced in 1945 by Lowenstein and Foster, who reported a deep, parallel split between the anterior and posterior stromal layers of the iris [2]. However, the condition was first described as early as in 1922 by Schmitt who reported on the detachment of the anterior iris layer [3].

## 4. Epidemiology and Inheritance

Only about 150 cases of iridoschisis have been reported to this date, with a slight predominance of female patients over males [4].

The question whether iridoschisis is a hereditary condition or not still raises controversies. Genetic background of this condition was first postulated by Danias et al. who reported on a mother with glaucoma and iridoschisis and her asymptomatic daughter without glaucoma, but with less extensive changes in the iris documented by high-frequency ultrasound imaging [5]. Further, Mansour described a family history of iridoschisis, anterior chamber shallowing and presenile cataract. The authors mentioned above speculated that iridoschisis might be inherited in an autosomal dominant manner, but these reports are scarce [4]. However, some researchers suggested that iridoschisis is not inherited but occurs sporadically, secondary to trauma, glaucoma or syphilis [2,6,7,8,9]. Other authors considered iridoschisis age-related atrophy, given that the condition is found predominantly in persons between 60 and 70 years of age [10]. As reviewed by Mansour, the mean and median ages of 131 patients with iridoschisis were 57 years and 62 years, respectively. Associated ocular findings including glaucoma, narrow angle, presenile cataract, eye trauma, cornea guttata or iris-corneal touch were found in 41%, 27%, 13%, 10%, 5% and 5% of patients, respectively [4]. However, it needs to be stressed that iridoschisis has also been reported in youths [11,12].

## 5. Pathophysiology

A number of theories exist regarding the pathogenesis of iridoschisis, but the exact underlying mechanism of this condition is yet to be explained.

Histopathological studies of the iris from iridoschisis patients documented tissue fibrosis and atrophy that lead to the formation of gaps between the anterior and posterior stromal layers [13].

Lowenstein and Foster suggested that iridoschisis is a consequence of either senile changes or blunt trauma or develops secondarily to glaucoma. They speculated that blunt trauma forced aqueous humor into the iris tissue destroying stroma with concomitant pigment dispersion [2,6]. Fluoroiridographic studies showed that in the affected sectors, blood flow in the vessels of the inner pupillary margin to the outer iris is normal, which implies that ischemia is unlikely a contributing factor [14]. However, some authors postulated that enhanced sclerosis of the irideal blood vessels might predispose anterior and posterior stromal layers to tear apart during dilation and constriction of the iris sphincter. Others suggested that the pathological changes observed in iridoschisis might be a consequence of an obliterative process [13]. In Bojer’s opinion, iridoschisis can be a form of essential iris atrophy in the elderly [15]. According to another hypothesis, prolonged treatment of glaucoma with miotic agents may evoke a mechanical shearing effect, which leads to tearing of the irideal stroma and resultant iridoschisis [16]. However, the latter theory is not necessarily correct, given that the miotics were used quite commonly in the past, which was not reflected by an increase in the prevalence of iridoschisis.

## 6. Clinical Characteristics and Diagnostic Imaging

While iridoschisis may be unilateral at its early stages, it occurs bilaterally in most cases [11] and may have a progressive character [17].

**Figure 2:**
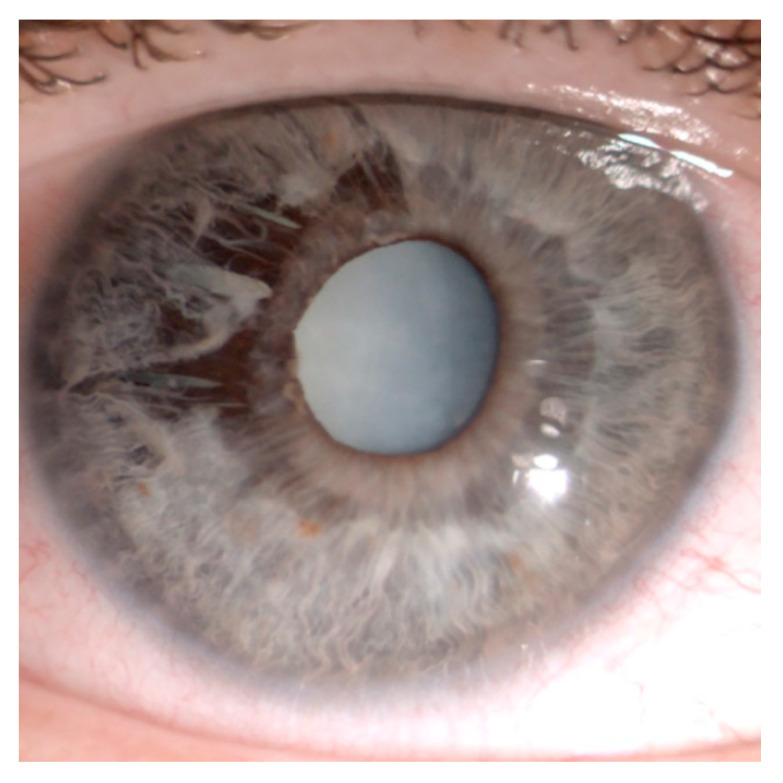
“Shredded” appearance of the iris: Superotemporal iridoschisis and mature cataract. Adapted from “Iris-claw lens implantation in a patient with iridoschisis” by Pieklarz B, Grochowski E, Dmuchowska DA, Saeed E, Sidorczuk P, Mariak Z. Am J Case Rep, 2020; 21: e925234.

The condition is most often found in the inferior irideal quadrants, but also other parts of the iris may be affected, and sometimes the pathological process is spread across the whole iris [1,4,18,19]. According to Mansour, the most common location of the pathological process in the group of 131 patients with iridoschisis were inferior irideal quadrants (37%), followed by the inferonasal (18%), superior (9%) and inferotemporal quadrants (7%); 29% of the patients presented with diffuse iridoschisis [4]. The anterior iris stroma splits from the posterior stroma and muscle layers, and the loose ends wave in the aqueous humor of the anterior chamber, giving the iris a “shredded” appearance. (Figure 2) The posterior layer of the iris usually remains intact with the retained function of the sphincter and dilator fibers [13].

Corneal changes are uncommon and, if present, the degenerated corneal endothelial cells are mostly localized above the area of iridoschisis [20,21,22,23,24,25]. Visual deterioration is usually caused by glaucoma, cataract or corneal decompensation secondary to iridocorneal touch [26].

**Figure 3:**
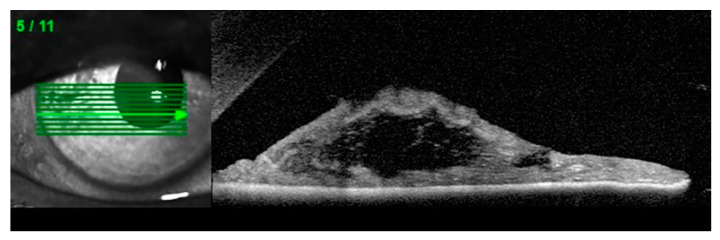
Anterior segment optical coherence tomography (AS-OCT): Disorganization of the iris stroma corresponding to iridoschisis (own source).

Anterior segment optical coherence tomography (AS-OCT), ultrasound biomicroscopy (UBM) and Scheimpflug imaging are complementary diagnostic options in patients with suspected iridoschisis. (Figure 3) [10,27,28]

An unquestioned advantage of AS-OCT and UBM over Sheimpflug imaging stems from the fact that the former two are suitable for direct visualization of the angle, ciliary body and sulcus [29]. While the abovementioned imaging methods are not essential to establish the diagnosis of iridoschisis, they may add to gonioscopy in the assessment of the iridocorneal angle. Furthermore, according to some authors, UBM should be carried out in patients in whom iridoschisis is associated with angle-closure glaucoma (ACG), to differentiate between the pupillary block and plateau iris configuration [28].

## 7. Glaucoma and Other Associated Ocular Pathologies

Iridoschisis may coexist with an array of other ocular pathologies, and in most of the cases, it is unclear whether it occurs as a cause or effect or just by coincidence.

Glaucoma, primarily angle-closure glaucoma, is found in more than two-thirds of patients with iridoschisis [1]. A coexistence of iridoschisis with chronic open-angle glaucoma or angle recession glaucoma has been reported as well [9,19,30]. Auffarth et al. presented the case of bilateral iridoschisis coexisting with pseudoexfoliation syndrome [31]. According to one published report, iridoschisis may also coexist with capsular delamination (true exfoliation) [32]. Salmon and Murray examined 12 patients with iridoschisis and coexistent primary angle-closure glaucoma. The authors concluded that iridoschisis is an unusual manifestation of iris stromal atrophy, and results from an intermittent or acute increase in intraocular pressure [19]. However, Romano et al. analyzed six cases of iridoschisis associated with ACG and found that the former preceded the angle closure episode or at least was not its consequence [7]. Nevertheless, the results of the studies mentioned above highlight the importance of excluding primary ACG in patients presenting with iridoschisis [9].

Some published evidence suggests that iridoschisis may coexist with presenile cataract and mature cataract [1,12,33,34,35].

While rarely, iridoschisis may also be found concomitantly to lens subluxation [7,19,33,36,37,38]. Agrawal et al. reported the case of unilateral iridoschisis coexisting with ipsilateral lens subluxation. According to those authors, mechanical factors, such as lens displacement, may contribute to iridoschisis by pushing the iris forward [36]. Mutoh et al. presented the case of lens displacement into the vitreous cavity associated with ipsilateral iridoschisis. In that report, iridoschisis was associated with periocular eczema [38]. A coexistence of iridoschisis with periocular eczema has also been mentioned by other authors [36]. However, Adler and Weinberg reported on a patient with bilateral lens subluxation and unilateral iridoschisis, which puts into question the mechanical etiologic theory mentioned above [37]. Thus, we still do not have enough evidence to state whether the contact of the iris with a subluxated lens might contribute to iridoschisis or if iridoschisis is associated with zonular abnormalities that eventually result in lens subluxation.

A rare combination of iridoschisis with keratoconus has been reported as well [39,40]. The research on potential common mechanisms of these two entities suggested possible genetic predisposition. Since the posterior layers of the cornea and the iris stroma share a common embryological origin, the coexistence of keratoconus with iridoschisis may point to inter-related pathogenesis [39]. Petrovic and Kymionis reported on a patient with bilateral keratoconus who developed massive iridoschisis involving the visual axis after four penetrating keratoplasties. The patient was successfully treated with Nd:YAG punctures to obtain an iris-graft detachment [40]. Another group reported on iridoschisis in conjunction with keratoconus and compulsive eye rubbing. The authors of that report hypothesized that chronic eye rubbing might affect the iris through mechanical trauma and intraocular pressure spikes, which eventually contributed to the development of iridoschisis. Moreover, it is well known that keratoconus is strongly associated with habitual eye rubbing [41].

Corneal abnormalities secondary to iridoschisis are uncommon. Iridoschisis may be a cause of focal corneal endothelial cell loss which can be detected in specular microscopy [42]. To the best of our knowledge, there are only a few published cases of localized bullous keratopathy secondary to iridoschisis. Free floating of iris fibers was shown to result in iridocorneal touch and subsequent local endothelial decompensation [20,22,23,24,25]. Total endothelial decompensation has been reported as well [21]. In one of the cases mentioned above, iridoschisis coexisting with bullous keratopathy was found in degenerative myopic eyes. While the presence of these two conditions in a single patient might be a coincidence, the authors of that case report did not exclude the involvement of hereditary factors [22]. In another published case report, iridoschisis and bullous keratopathy were associated with nanophthalmos and microcornea [24]. However, some other authors described iridocorneal touch due to iridoschisis without resultant corneal changes [5,7,27,28].

We found one published report on Marfan syndrome concomitant to iridoschisis [7]. Coexistence of iridoschisis with congenital syphilis with or without concomitant interstitial keratitis has been reported as well. According to some authors, the pathogenesis of iridoschisis secondary to syphilis might involve immunological factors [8,9].

The authors of another interesting published case report described a patient with simultaneous bilateral nonarteritic anterior ischemic optic neuropathy (NAION) associated with acute ACG secondary to iridoschisis. According to the authors, elevated intraocular pressure might be the main precipitating factor for the development of NAION in their patient [26]. Iridoschisis can also coexist with plateau iris configuration [28,43]. We also found one case report on juvenile iridoschisis with incomplete plateau iris configuration and without evidence of glaucoma [27]. Swaminathan et al. reported on a patient in whom assault with an alkaline substance resulted in bilateral chemical keratoconjunctival, trabecular and lenticular damage; additionally, the patient presented with unilateral iridoschisis [44].

## 8. Differential Diagnoses

The diagnosis of iridoschisis is based on slit lamp examination that shows the characteristic appearance of the iris. The differential diagnoses of iridoschisis include two other principal stromal anomalies of the iris, iridocorneal endothelial (ICE) syndrome and Axenfeld–Rieger syndrome (ARS).

ICE syndrome is a group of disorders associated with the presence of abnormal corneal endothelium, accompanied by iris atrophy of various degree, secondary ACG, corneal edema and pupillary anomalies. Three clinical variants of the ICE syndrome have been described to this date: Chandler syndrome, essential iris atrophy and Cogan-Reese syndrome.

ARS is associated with anterior segment dysgenesis and systemic abnormalities. The ocular findings are typically present at birth, but progressive changes in the iris and angle defects may also be detected later in childhood. The variants of this condition include Axenfeld anomaly (prominent, anteriorly displaced Schwalbe line, with associated iridocorneal adhesions), Rieger anomaly (manifestations specific for Axenfeld anomaly plus central irideal changes) and Rieger syndrome (all ocular findings typical for Rieger anomaly plus nonocular features) [45,46].

Principal differences between the entities mentioned above and iridoschisis are summarized in Table 1.

## 9. Glaucoma Characteristics and Treatment in Patients with Iridoschisis

Angle-closure glaucoma or angle narrowing resulting in an acute angle closure associated with iridoschisis are usually treated with peripheral laser iridotomy to relieve the pupillary block [5,19,20,26,28,47,48]. However, some authors suggested that goniosynechialysis combined with cataract removal is superior to laser peripheral iridotomy in patients with iridoschisis complicated with closed-angle glaucoma triggered by peripheral anterior synechiae [47]. In turn, Porteous et al. described the uneventful treatment of two patients with acute angle-closure glaucoma and concomitant iridoschisis with lens extraction and IOL implantation. Postoperative IOPs in those two patients remained within normal limits without topical medication, and the angles were open on gonioscopy [35]. Finally, Shima et al. suggested that UBM should be performed whenever angle-closure glaucoma coexists with iridoschisis to differentiate between the pupillary block and plateau iris configuration, and postulated that combined trabeculotomy and cataract surgery could be a useful treatment option in persons in whom glaucoma occurs concomitantly to iridoschisis associated with plateau iris configuration [28].

Moreover, the applicability of other antiglaucoma procedures, such as trabeculectomy [9,19], implant surgery (Molteno tube) [19] or iridectomy and cataract extraction with intraocular lens implantation as primary surgical treatment [19,23] has been reported in the literature.

Clinical characteristics, presentation and treatment of glaucoma concomitant to iridoschisis are summarized in Table 2.

## 10. Phacoemulsification in Patients with Iridoschisis

Management of iridoschisis during cataract surgery or other anterior chamber procedures may be challenging. Snyder et al. highlighted potential risks and complications during and after the cataract surgery. First, the iris fibrils may be aspirated by the phaco probe or irrigation-aspiration probe. Second, the exposure of pigment epithelium of the iris may predispose to symptomatic photic phenomena. Third, the sphincter muscle can be damaged [49]. Lastly, according to some authors, the phacoemulsification procedure can be more challenging because of poorly dilating pupils [23,47,49,50].

According to some authors, the intraoperative circumstances during cataract procedure in a person with iridoschisis may resemble those in a patient with the floppy iris syndrome [47]. The surgery can be performed without pupil expander devices [18]. However, additional precautionary measures may be undertaken to increase the safety of the procedure. One approach is to use a dispersive viscoelastic, e.g., 3% sodium hyaluronate, to hold the iris fibrils in place [18,51]. However, it should be remembered that the viscoelastic is gradually removed with the phaco probe or aspiration-irrigation probe, and thus needs to be reinjected periodically.

Our own experiences suggest that patients with small pupils may require additional maneuvers. In such patients, the pupil can be gently stretched with two spatulas. During chopping and removal of the affected quadrant, a chopper can help move the pupil margin sideways, and an irrigation needle may play the same function during the irrigation-aspiration stage. Whichever method is used, all manipulations should be careful and limited solely to the pupillary center, and minimum required fluidic parameters need to be maintained.

Another approach is the use of iris hooks (retractors) or pupil expanders, such as Malyugin ring, Greather pupil expander or the Perfect Pupil Iris Extension System [23,31,48,52,53]. Other techniques reported in the literature involve the excision of floating iris fibers with a vitreous cutter or Vannas scissors [10,50,54]. Recently, a novel approach to the free fibril management has been proposed by Snyder et al. They applied a microcautery causing collagen contraction, shrinking the cords back to the iris surface, without the removal of any structural components [49].

**Figure 4:**
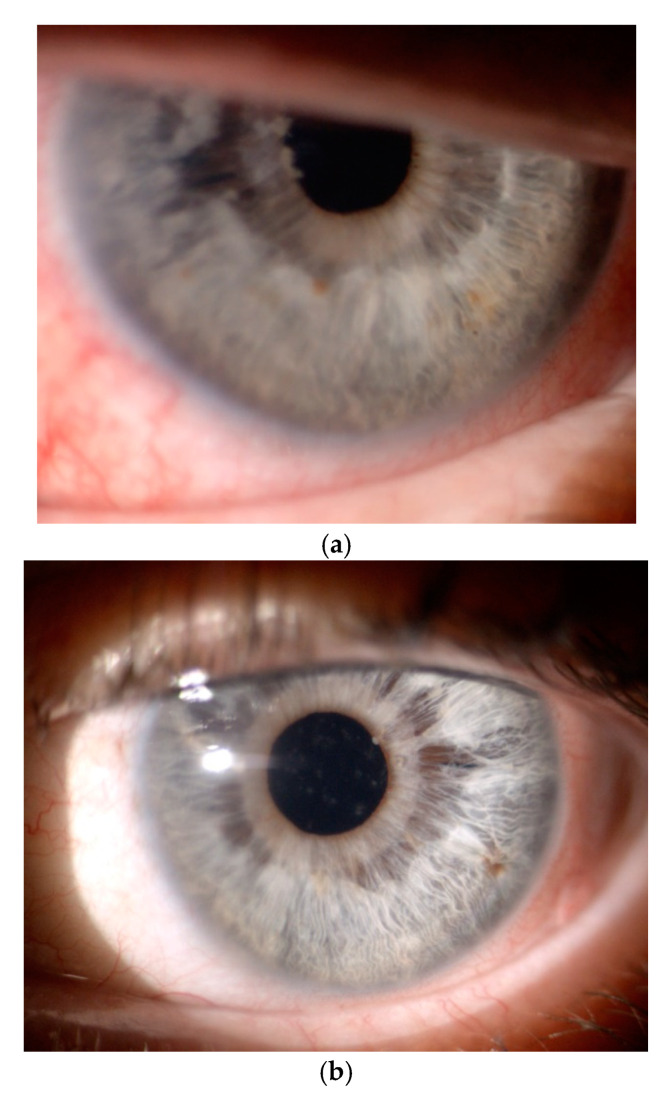
Postoperative findings in a patient with bilateral iridoschisis, associated lens subluxation, mature cataract and secondary glaucoma treated in our department. (**a**) Right eye following vitrectomy with lensectomy and intrascleral sutureless intraocular lens fixation (Yamane technique) on the first postoperative day. (**b**) Left eye following vitrectomy with lensectomy and iris-claw lens.

The course and outcomes of cataract surgeries in patients with iridoschisis can be negatively affected by concomitant zonulopathy with lens subluxation or luxation and bullous keratopathy [36,37,38]. In persons with iridoschisis, aphakia should be treated by implantation of scleral fixated lenses, rather than iris-claw lenses [33] (Figure 4) and all additional risks should be listed in the informed consent form.

## 11. Corneal Decompensation Treatment in Patients with Iridoschisis

We found two published reports documenting the use of endothelial keratoplasty in the treatment of corneal decompensation in patients with iridoschisis [23,25]. Minezaki et al. reported on a patient with bullous keratopathy secondary to iridoschisis treated by non-Descemet’s stripping automated endothelial keratoplasty (nDSAEK). The nDSAEK was carried out four days after phacoemulsification and iridectomy. Postoperative best corrected visual acuity (BCVA) in the patient improved to 6/6 [23]. In turn, Greenwald et al. performed bilateral cataract extraction and superficial iridectomy followed by Descemet membrane endothelial keratoplasty (DMEK), achieving postoperative improvement of BCVA in both eyes to 6/6 [25].

Another treatment modality described in the literature is penetrating keratoplasty (PK) combined with cataract extraction [9,19,20,21].

Other authors described amniotic membrane transplantation as a treatment to relieve pain caused by bullous keratopathy in a patient with iridoschisis. The patient also presented with nanophthalmos, microcornea, shallowing of the anterior chamber and anterior and posterior scleral thickening. In that case, PK was considered a high-risk procedure because of the potential risk of a synechial angle closure [24].

Wesseley and Freeman suggested that iridectomy might be considered a prophylactic approach to remove disrupted iris fibers, as the latter may play a role in the development of corneal changes in selected patients [42].

## 12. Discussion

The prevalence of iridoschisis is difficult to estimate. It is probably underreported as no dedicated registry for this condition exists.

As shown in this review, some questions regarding iridoschisis are yet to be explained. It is still unclear whether this condition is hereditary or sporadic. Further, more consistent information about the pathophysiology of iridoschisis is required to develop an effective treatment to prevent or slow down the progression of this condition. Moreover, questions arise regarding the surgical approach to cataract in patients with concomitant iridoschisis. While a plethora of various methods have been used to manage iris fibrils and small pupil, we still lack information about the most suitable artificial lens type and material. Similarly, little is known about a preferable lens fixation method in cases with deficient capsular support and published reports on such treatment of aphakia in iridoschisis patients are scarce.

## 13. Conclusions

Iridoschisis is plausibly a multifactorial disease that requires particular attention from ophthalmologists and ophthalmic surgeons.

A patient diagnosed with iridoschisis should also be screened for potential glaucoma, corneal and lens abnormalities. If not yet present, one of these conditions may subsequently develop, and hence iridoschisis patients should be followed-up on a regular basis.

Ophthalmic surgeons may expect problems during and after cataract surgery in a patient with iridoschisis. Therefore, an informed consent form, listing all additional potential complications, should be signed by each patient. The perioperative risks can be mitigated with an array of methods.

## Figures and Tables

**Table 1:** Differential diagnoses of iridoschisis. Principal characteristics and distinguishing features of iridoschisis, iridocorneal endothelial (ICE) syndrome and Axenfeld–Rieger syndrome (ARS) [4,11,43,45,46].

Distinguishing Features	Iridoschisis	ICE			ARS
		Chandler Syndrome	Cogan-Reese Syndrome	Essential Iris Atrophy	
**Age of Onset**	6th–7th decade	3rd–4th decade			congenital, at birth
**Gender Predisposition**	slightly more often in women	mostly in women			no
**Laterality**	more often bilateral	mostly unilateral			bilateral
**Congenital**	no	no			yes
**Iris**	- stromal splitting with strands floating in the aqueous humor - sectoral, mainly inferior - without hole(s)	- minimal irideal alterations - areas of irideal atrophy should not progress to a full-thickness iris hole(s)	- different degrees of iris atrophy - multiple yellowish or brown irideal nodules	- irideal atrophy - stretched and full-thickness hole(s)	- iris may be normal except for the iridocorneal adhesions - mild stromal thinning - atrophy with full-thickness holes - stromal hypoplasia
**Localization**	mostly inferior, but also other parts of the iris may be affected	diffuse			diffuse
**Cornea**	rarely involved, but focal endothelial cell loss may occur, possibly with subsequent endothelial decompensation	- evident early corneal edema	- corneal edema may occur	- corneal edema may occur	- central cornea usually normal - endothelial changes due to PAS - edema - posterior embryotoxon
		- endothelial dystrophy - specular microscopic evidence of ICE-cells, i.e., abnormal, rounded, large and pleomorphic cells with pathognomonic intracellular dark spots			
**Pupils**	round and reactive	corectopia	changes uncommon	polycoria	- corectopia - polycoria - ectropion uvae
**Anterior Chamber Angle**	PAS possible	gradually progressing PAS			PAS
**Progression**	yes	yes			often stationary or only a minor progression
**Glaucoma**	- various forms of glaucoma in 65% of the cases: - most often secondary ACG (Table 2) - sporadically open-angle glaucoma [9,14], angle recession glaucoma [30]	secondary ACG in 50% of the cases			secondary ACG in 50% of the cases
**Other Ocular or Systemic Anomalies**	a number of possible associated ocular conditions (uncertain relevance)	no non-ocular findings			- craniofacial dysmorphism-dental abnormalities-musculoskeletal malformations - possibility of other systemic abnormalities

Abbreviations: PAS—peripheral anterior synechiae; ACG—angle closure glaucoma.

**Table 2:** Clinical characteristics and anti-glaucoma treatment in patients with iridoschisis and concomitant glaucoma. Reports on iridoschisis without concomitant glaucoma or with insufficient information are not included. Some data are missing due to insufficient information provided in the source papers.

Author, Publication Year, Reference	Type of Glaucoma	Age (Years), Sex	History of Ocular Trauma or Hereditary Ocular Disease	Clinical Characteristics and Imaging Results	Iridoschisis Laterality, Localization	Other Findings	Conservative Treatment	Surgery or Laser Treatment	Comments
**Gogaki et al., 2011 [1]**	AAC (ACG possible)	80, F	No	- VA: LE 6/200 - IOP: LE 54 mmHg - Biomicroscopy: typical findings in AC - Optic disc: evaluation impossible - Gonioscopy: closed angle (Shaffer grade 0-1) for 270° and scattered PAS in the superior quadrants of both eyes	Bilateral, inferiorly	Mature cataract	- Acute treatment: acetazolamide, dorzolamide/timolol, isoptocarpine, intravenous mannitol and oral analgesics - Chronic treatment: isoptocarpine	No	Good IOP control with topical treatment
**Mansour, 1985 [4]**	Case 1: likely ACG in LE	54, F	No information	- BVCA: RE 6/60, LE 6/2000 - IOP: RE 10 mmHg, LE 12 mmHg - Optic disc: no data - Gonioscopy: RE open anterior chamber angle with normal angle structures for 360°, LE anterior chamber +1 centrally and peripherally with grade 1 to 2 angle - Provocative test: increase in IOP to 13 mmHg	Bilateral, inferiorly, more prominent in LE	- Pseudophakic RE (lens extraction 3 years earlier) - Dense nuclear cataract in LE	No	Uncomplicated intracapsular lens extraction and iridectomy in LE	Postoperative BVCA: LE 6/30
	Case 2: ACG	57, F (sister of patient from Case 1)	No information	- VA: RE 6/600, LE hand motion - IOP: RE 14 mmHg, LE 4 mmHg - Optic disc: no data - Gonioscopy: RE narrow angle with no structures seen and PAS superiorly	Extensive, bilateral iridoschisis	- Cataract in RE - Iris incarceration, pupillary occlusion and phthisis bulbi with low intraocular pressure (post-operative complications) in LE	No	- Extracapsular lens extraction in RE - Complicated intracapsular lens extraction in LE four years earlier	- Postoperative BVCA: RE 6/21
**Danias et al., 1966 [5]**	ACG	71, F	History of the excision of fibrous dysplasia from the left frontal bone and superior orbital decompression	- BVCA: RE 6/7.5, LE 6/12 - IOP: RE 23 mmHg, LE 26 mmHg - Optic disc: RE c/d = 0.5, LE c/d = 0.8 - Gonioscopy: narrow angles - Goldmann perimetry: full fields in both eyes although the Humphrey hemifield borderline glaucomatous in LE	Bilateral, all quadrants affected, more pronounced inferiorly and in LE	- Iridocorneal touch inferiorly in RE - Nanophthalmos - Hyaloid residues in LE	No information	Laser iridotomy in LE	IOP one month later: LE 23 mmHg with essentially unchanged gonioscopic findings
**Romano et al., 1972 [7]**	Case 1: ACG	63, M	No information	- VA: RE 6/76, LE 6/76 - IOP: RE 14 mmHg, LE 13 mmHg - Optic discs: partial cupping - Gonioscopy: PAS in the lower in both eyes	Bilateral, symmetrical, between 4 and 7 o’clock position	Bilateral nuclear and slight posterior cortical cataract	Pilocarpine and acetazolamide	- Bilateral basal iridectomy 17 years earlier because of AAC - One year later uneventful intracapsular cryo-extraction of cataract in RE	- No obvious progression of iridoschisis during a three-year follow-up - Postoperative BCVA in RE: 6/24
	Case 2: ACG	61, F	No information	- VA: RE 6/8, LE 6/10 - IOP: RE 14 mmHg, LE 40 mm Hg - Optic disc: RE “normal”, LE “almost completely excavated” - Gonioscopy: narrow angle in LE, closed in the lower quadrants - Provocative test with neosynephrine: IOP raised to 24 mm in RE	Unilateral in RE, between 4 and 7 o’clock position		Pilocarpine, eserine into LE, pilocarpine into RE, acetazolamide	- Peripheral iridencleisis in LE - Peripheral iridectomy in RE (AAC in the night after the LE surgery)	Minor progression of iridoschisis during a two-year follow-up
	Case 3: ACG, AAC (3 weeks earlier)	75, F	No information	- VA: “normal” in both eyes - IOP: RE >30 mmHg in spite of intensive treatment, LE 18 mmHg - Optic discs: no cupping - Gonioscopy: almost completely closed angle in RE, very narrow but still open angle in LE - Visual field: upper nasal defect in RE - Provocative test: no increase in IOP in LE	Bilateral, between 5 and 7 o’clock position	Iridocorneal touch	Pilocarpine in both eyes	- Iridencleisis in RE	
	Case 4: ACG in RE	55, F	No information	- VA, IOP, optic disc: no data - Gonioscopy: closed angle except from the area of the coloboma - Patient under control for the last 15 years; lost vision in LE	Between 4 and 8 o’clock position	Nuclear posterior cortical cataract in RE (developed later)	No information	- Total iridectomy in RE (development of a spontaneous filtrating bleb with further loss of activity) - Cyclodialysis in RE two years later (tension could not be controlled) - Cataract; extraction of the lens in RE	
**Foss et al., 1992 [9]**	Case 1 and 3: no evidence of glaucoma								
	Case 2: 10-year history of bilateral chronic open angle glaucoma	70, F	No information	- VA: RE 6/24, LE 6/20 - IOP: 18 mmHg in both eyes - Optic disc: glaucomatous damage in both eyes - Gonioscopy: a limited view of the open angle with moderate pigment deposition, without PAS - Visual fields: constrictions in both eyes	Bilateral	Bilateral interstitial keratitis	Topical antiglaucoma treatment	No	No progression on topical antiglaucoma treatment
	Case 4: 24-year history of bilateral chronic open angle glaucoma	75, F	No information	- VA: RE hand movements, LE 6/60 - IOP: RE 16 mmHg, LE 5 mmHg - Optic disc: c/d = 0.5 in both eyes - Gonioscopy: open anterior chamber angles with mild pigment deposition	Unilateral, inferiorly	- Interstitial keratitis and old keratic precipitates in LE - Bilateral inferonasal chorioretinal scarring - Moderate nuclear cataract in RE	Topical antiglaucoma medication for 10 years, followed by IOP increase to 50 mmHg (despite topical and oral acetazolamide)	- Uncomplicated trabeculectomies in both eyes (no additional medication needed for 14 years after the surgery) - Extracapsular cataract extraction and penetrating keratoplasty in RE 10 years after the first surgery	
**Chen et al., 2017 [10]**	Case 1 and 2: no evidence of glaucoma								
	Case 3: ACG	66, M	No	- Preoperative BCVA: RE 6/38, LE 6/15 - IOP: RE 22 mmHg, LE 35 mmHg - Optic disc: no data - Gonioscopy: Discontinuous PAS and pigment deposition superiorly - Scheimpflug images: peripheral AC partially shallowed, but the iridocorneal angle still open in both eyes	Bilateral, temporally	Cortical cataract and dust turbidity of the vitreous body	- Travoprost, brinzolamide, carteolol, brimonidine - On topical medication–IOP: RE 18 mmHg, LE 32 mmHg	Glaucoma surgery recommended as a necessary treatment option in the future	Significant fluctuations of IOP with pharmacotherapy
**Carnevalini et al., 1988 [14]**	Case 1: no evidence of glaucoma								
	Case 2: open angle glaucoma	66, M	No	- VA/Optic disc: no data - IOP: 12 mmHg in both eyes	Unilateral, inferiorly in RE	No data	Pilocarpine	Trabeculectomy five years earlier	
	Case 3: glaucoma in RE (diagnosed after head trauma)	55, F	Head trauma 20 years earlier	- VA: RE 6/600 - IOP: RE 14 mmHg, LE 10 mmHg - Optic disc: post-traumatic atrophy in LE	No data	Post-traumatic optic nerve atrophy in LE	Miotics in RE		
	Case 4: no data (abstract only)								
**Payne and Thomas, 1966 [16]**		79, M	No information	- VA: RE 6/30, LE hand movements - IOP: RE 20 mmHg, LE 35 mmHg (before treatment), later 20 mmHg in both eyes - Optic disc: severe cupping and atrophy in both eyes - Gonioscopy: chamber angles open albeit narrow - Visual fields: 5 to 10 degrees in both eyes	- Bilateral, inferiorly - Iridoschisis following miotic therapy,	Bilateral subcapsular cataract	Pilocarpine and, eventually, epinephrine bitartrate	No	Glaucoma readily controlled with epinephrine bitartrate (as stated by the authors)
**Salmon and Murray, 1992 [19]**	12 patients (summary) AAC or ACG	3 M, aged 54-72 9 F, aged 39-76	No	- IOP: mean 53 mmHg (range 12-65 mmHg) - In 10 patients with a history of headaches or ocular pain: a. optic discs: evidence of glaucomatous disc damage with a corresponding field defect in 14 eyes from seven patients; no damage to the optic nerve in five eyes from three patients b. gonioscopy: evidence of angle closure, in particular involving the superior angle in all ten patients - In two patients without headaches or ocular pain: a. AAC after pupillary dilation in one case b. closed superior angle with normal optic disc in another case	- Bilateral in 7 patients - Unilateral in 5 patients - Inferiorly in 17 eyes - Superiorly and inferiorly in 2 eyes	- Limited corneal touch in 12 eyes: localized corneal opacity in one eye, decompensated, edematous cornea in one eye, interstitial keratitis in one patient - Clear lenses in two eyes - Lens subluxation in one patient - Nuclear or cortical cataract in all remaining eyes; in some with anterior subcapsular lens opacities (suggestive of previous Glaukomflecken)	- Preoperative topical treatment in all patients (no more information)	- YAG laser peripheral iridotomy in fourteen eyes - Trabeculectomy in three patients after iridotomy - Uniocular implantation of a Molteno tube in one patient despite the treatment mentioned above (persistent increase in intraocular pressure) - Cataract extraction with intraocular lens implantation and iridectomy as primary surgical treatment in two patients (corneal graft simultaneously to cataract extraction in one eye) - Subsequent cataract surgery in further five patients during a five-year follow-up period - Enucleation in one eye (untreated AAC)	Long-term intraocular pressure control was achieved in all patients, although topical pilocarpine was required after peripheral iridotomy in four cases (as stated by the authors)
**Rodrigues et al., 1983 [20]**	Case 1: increased IOP in RE	87, F	No information	- VA: RE 6/20, LE 6/60 - IOP: RE 24 mmHg, LE 14 mmHg - Optic disc: normal in both eyes - Gonioscopy: open angle (Schaffer grade 2-3 wherever the angle could be visualized	Bilateral, inferiorly	- Iridocorneal touch in both eyes - Bullous keratopathy in LE - Mild bilateral senile macular degeneration	No	Combined corneal transplant, cataract extraction and large sector iridectomy inferiorly in LE	
	Case 3	63, F	No information	- VA: no data - IOP: 22 mmHg in both eyes - Optic disc: early glaucomatous damage in both eyes - Progressive visual field loss - Gonioscopy: narrow anterior chamber angles	Bilateral, inferiorly	Bilateral early cataract	- Pilocarpine and acetazolamide –> increase in IOP to >30 mmHg despite the addition of epinephrine (as stated by the authors)	- Trabeculectomy followed by cataract extraction in LE - Cataract extraction with a wide iridectomy in RE	- unsatisfactory visual result after cataract extraction in LE due to the presence of a friable material from the iris over the anterior vitreous face with occlusion of the pupillary aperture -> vitrectomy with removal of the iris material - Postoperative VA: RE 6/10, LE 6/15, postoperative IOP: RE 15 mmHg, LE 31 mmHg
	Case 4	51, F	No information	- VA: RE 6/60 -> then, light perception, LE light perception - IOP: RE 24 mmHg, LE 25 mmHg - Optic disc: advanced glaucomatous damage - Gonioscopy: no data	Probably bilateral	Cataract	Pilocarpine and acetazolamide	Filtration surgery	
	Case 5	72, F		- VA: RE 6/20, LE light perception with projection - IOP: RE 16 mmHg, LE 19 mmHg - Optic disc: RE normal, LE could not be visualized - Gonioscopy: AC angle in RE optically closed secondary to strings of iris stroma; angle slit-like albeit open between the 3 and 6 o’clock positions - AC angle narrow and optically closed superonasally, no definite PAS	Bilateral, inferonasally	Mature cataract in LE and mild cataract in RE	No	Cataract extraction and sector iridectomy in LE	
	Case 6 ACG	65, M	No information	- VA: RE 6/12, LE 6/60 - IOP: RE 8 mm Hg, LE 17 mm Hg - Optic disc: RE c/d = 0.3, LE not visible because of the marked AC reaction - Gonioscopy: narrow AC angle in RE, optically closed AC angle in LE with PAS superiorly	- Bilateral, inferotemporally in RE - More marked in LE: inferiorly, in particular nasally	- Corneal edema and inflammatory reaction (including fibrin, 3+ cells and flare in LE)	- Pilocarpine postoperatively to LE	- Surgical peripheral iridectomy in RE - Laser iridectomy in LE	- Postoperative: IOP in RE 15 mmHg without topical treatment, laser iridectomy closure in LE -> IOP 19 mmHg -> pilocarpine -> peripheral iridectomy inferiorly in the area of the iridoschisis - Final postoperative VA 6/12 in both eyes, Final IOP: RE 16 mmHg, LE 19 mmHg without therapy
	Case 2: no evidence of glaucoma								
**Minezaki, 2013 [23]**	ACG	79, F	No information	- BCVA: RE hand movements, LE 6/8 - IOP: RE not determined, LE 8 mmHg		- Bullous keratopathy - Virtually nonexistent anterior chamber in RE		- Cataract surgery with iridectomy succeeded to deepen the anterior chamber and to remove the floating iris leaf, although corneal edema remained - NDSAEK four days later resolved corneal edema and restored visual acuity	
**Torricelli et al., 2011 [26]**	ACG	55, F	No information	- VA: RE hand movements, LE light perception - IOP: RE 42 mmHg, LE 44 mmHg - Optic disc: edema with no vascular tortuosity and no cup in both eyes - Gonioscopy: narrow angles (Shaffer grade 0)	Bilateral, inferiorly	NAION	Timolol, brimonidine, pilocarpine, prednisolone eye drops, mannitol and acetazolamide	YAG laser iridotomy provided satisfactory IOP control	- Two months later: BCVA RE 6/6, LE 6/60; IOP 11 mmHg in both eyes with no medication; optic disc edema followed by optic disc pallor in both eyes (as stated by the authors)
**Paniagua et al., 2015 [27]**	Iris plateau	80, F	No	- VA: RE <6/60, LE 6/15 - IOP: 14 mmHg in both eyes - Optic disc: no data - Gonioscopy: slight angle closure (Shaffer grade 2 in both eyes), no PAS - Ultrasonic biomicroscopy: incomplete angle closure in both eyes in all four quadrants caused by the anterior displacement of the ciliary body (iris plateau)	Bilateral, inferonasal quadrants	- Iridocorneal contact - Cortical cataract	No information	No information	
**Shima et al., 2007 [28]**	ACG	79, M	No information	- VA: RE 6/6, LE 6/5 - IOP: 24 mmHg in both eyes - Optic disc: glaucomatous damage, RE c/d = 0.7, LE c/d = 0.8 - Gonioscopy: narrow angles (Shaffer grade 2) for 270° and scattered PAS in the superior quadrants in both eyes - Goldmann perimetry: temporal arcuate scotoma in both eyes - UBM: typical features of plateau iris configuration	Bilateral, inferiorly	Iridocorneal contact in the inferior quadrants of both eyes	Neither medical treatment with latanoprost, carteolol or pilocarpine eye drops nor laser gonioplasty provided satisfactory IOP control	Combined trabeculotomy and phacoemulsification in both eyes	Postoperative IOP: RE 13 mmHg, LE 11 mmHg with timolol treatment (four months after the surgery)
**Salmon, 1992 [30]**	Case 1: ARG	54, M	History of facial and ocular trauma at young age	- BCVA: RE 6/20, LE 6/30 - IOP: RE 57 mmHg, LE 58 mmHg - Optic disc: advanced glaucomatous damage in both eyes - Gonioscopy: angle recession 180° in RE and 90° in LE	Bilateral, superior-inferiorly	Corneal edema in RE	IOP was not controlled pharmacologically	- Trabeculectomy in both eyes - Molteno tube in LE, subsequently	Postoperative IOP controlled in both eyes with topical antiglaucoma treatment (four years later)
	Case 2: ARG	50, M	History of ocular trauma many years earlier	- VA: RE 6/12 - IOP: RE 40 mmHg - Optic disc: advanced glaucomatous damage in RE - Advanced visual field loss in RE - Gonioscopy: angle recession 110° in RE	Plausibly unilateral, superior-inferiorly	Phthisis in LE	IOP was not controlled pharmacologically	Trabeculectomy in RE	
**Pieklarz et al., 2020 [33]**	ACG	47, M with moderate intellectual disability, possible Marfan syndrome	No	- VA: hand motion in both eyes - IOP: RE 34 mmHg, LE 30 mmHg - Optic disc: RE c/d = 0.6, LE c/d = 0.9 (postoperatively)	Bilateral, superior-temporally	- Bilateral temporal lens subluxation - Bilateral mature cataract	Topical antiglaucoma treatment in both eyes	- Pars plana vitrectomy and lensectomy with iris-claw lens implantation in LE complicated by possible uveitis-glaucoma-hyphema syndrome - Uneventful pars plana vitrectomy with lensectomy and intrascleral sutureless intraocular lens fixation (Yamane technique) in RE	Postoperative VA: RE 6/60, LE 6/152; postoperative IOP: RE 17 mmHg, LE 29 mmHg
**Porteous et al., 2014 [35]**	Case 1: AAC in RE	49, M	No information	- BVCA: RE 6/12, LE 5/6 - IOP: RE 42 mmHg, LE 20 mmHg - AS-OCT: secondary angle closure in both eyes	Bilateral, superiorly and inferiorly	Nuclear cataract in RE	Oral acetazolamide and maximal topical IOP lowering medication in RE reduced IOP to 18 mmHg within 2 h	Uncomplicated cataract extraction in RE	Postoperative BCVA: RE 6/6; postoperative IOP: RE 18 mmHg without medication
	Case 2: AAC in LE	62, M	No information	- VA: LE 6/36 - IOP: LE 50 mmHg - Optic disc: LE normal - UBM: crowding of the inferior angle from iris strands	Unilateral, inferiorly in LE	Cataract in LE	- Oral acetazolamide and maximal topical IOP lowering medication reduced IOP within 2 h in LE - Prostaglandin analogue before the surgery	Uncomplicated cataract extraction in LE	Postoperative BCVA: LE 6/12; postoperative IOP: LE 14 mmHg without medication
**Agrawal et al., 2001 [36]**	ACG in LE; AAC in RE	50, M	No trauma	- BVCA: RE 6/6, LE 6/12 - IOP: RE 16 mmHg, LE 30 mmHg (despite medication) - Optic disc: normal in LE - Gonioscopy: slit angles in RE, closed angles with multiple PAS in LE - Visual field: normal in RE, generalized depression in LE	Unilateral in LE, inferotemporally;	Inferotemporal lens subluxation in LE	Local and systemic antiglaucoma medication in LE; pilocarpine in RE	- Trabeculectomy in LE - Intracapsular cataract extraction in LE six years later (advanced cataract)	- The patient did not consent for laser iridotomy; IOP and visual fields remained stable thereafter - Three years later, the patient developed AAC in RE as he had discontinued the pilocarpine eyedrops; he resumed to use of the drops - During a follow-up, the iridoschisis, present only in LE, remained unchanged
**Swaminathan et al., 2017 [44]**	Iridoschisis and glaucoma possibly secondary to alkali burn	F, in her early 30′s		Progressive increase in IOP	Probably secondary to alkali burn	- Cataract - Corneal opacification unresponsive to pharmacotherapy		A glaucoma drainage implant in LE without complications, with resultant improvement of IOP control	
**You et al., 2017 [47]**	Bilateral ACG	67, F	No	- VA: RE 6/30, LE light perception - IOP: RE 58 mmHg, LE 22 mmHg - Optic disc: RE c/d = 0.8, LE ocular fundus fuzzy - Gonioscopy: AC angle N IV (Scheie classification); PAS detected in 3 out of 4 quadrants via dynamic gonioscopy in RE and in all quadrants in LE	Bilateral, inferiorly	- Cortical cataract in both eyes - Pterygium in LE	- For financial reasons, the patient elected to postpone surgical treatment of LE - four IOP-lowering drugs: pilocarpine, timolol, brinzolamide, and brimonidine in LE	-Goniosynechialysis and phacoemulsification with IOL implantation in RE - Laser peripheral iridotomy in LE	- Postoperative VA: RE 20/33; postoperative IOP RE 11 mmHg - The outcome in this case suggests that laser peripheral iridectomy may provide limited treatment in iridoschisis complicated by ACG triggered by PAS; goniosynechialysis combined with cataract removal seems a better treatment option (as stated by the authors)
**Gomez Goyeneche et al., 2018 [48]**	Case 1	70, M	No	- VA: 6/6 in both eyes - IOP: 28 mmHg in both eyes - Optic disc: normal in both eyes - Gonioscopy: closed angle (Shaffer grade 0–1) for 270° in both eyes - Visual field: normal in both eyes	Unilateral, inferior-nasally in RE	Bilateral mild cataract	- An ocular hypotensive agent was initiated	The patient was scheduled for bilateral peripheral laser iridotomy	A 6-month follow-up was scheduled for glaucoma review
	Case 2: ACG	72, M	No	- BCVA: RE 6/24, LE CF - IOP: 12 mmHg in both eyes - Optic disc: c/d = 0.9 in both eyes - Gonioscopy: permeable iridotomy and Shaffer grade 3 angles in both eyes	Unilateral, inferior-nasally in LE	- Mature cataract in LE - Pseudophakic RE	No	- The patient was scheduled for trabeculectomy combined with cataract extraction in LE - Past history of iridotomy in RE	
	Case 3	59, F	No	- BVCA: 6/7.5 in both eyes - IOP: RE 14 mmHg, LE 16 mmHg - Optic disc: normal in both eyes - Gonioscopy: Shaffer grade 2angles	Bilateral, inferior-nasally	- Hyphema in RE - Bilateral mild cataract	No	The patient was scheduled for bilateral peripheral laser iridotomy	A 6-month follow-up was scheduled for glaucoma review

Abbreviations: M—male; F—female; VA—visual acuity; BCVA—best corrected visual acuity; ACG—angle closure glaucoma; AC—angle closure; ARG—angle recession glaucoma; RE—right eye; LE—left eye; IOP—intraocular pressure; PAS—peripheral anterior synechiae; AS-OCT—anterior segment optical coherence tomography; UBM—ultrabiomicroscopy; IOL—intraocular lens.
